# Disturbance of Social Hierarchy by an Invasive Species: A Gene Transcription Study

**DOI:** 10.1371/journal.pone.0002408

**Published:** 2008-06-11

**Authors:** Christian Roberge, Simon Blanchet, Julian J. Dodson, Helga Guderley, Louis Bernatchez

**Affiliations:** 1 Département de biologie, Université Laval, Québec, Canada; 2 Laboratoire Évolution et Diversité Biologique, C.N.R.S-Université Paul Sabatier, Toulouse, France; University of Uppsala, Sweden

## Abstract

**Background:**

Ecological and evolutionary changes in native populations facing invasion by exotic species are increasingly reported. Recently, it has been shown that competition with exotic rainbow trout (*Oncorhynchus mykiss*) disrupts dominance hierarchies within groups of native Atlantic salmon (*Salmo salar*). The genetic and molecular actors underlying phenotypic plasticity are poorly understood.

**Methodology:**

Here, we aimed at identifying the genetic and molecular actors contributing to this plastic loss of dominance hierarchies as well as at identifying genes implicated in behaviours related to social dominance. By using microarrays, we compared the genome-wide gene transcription profiles in brains of dominant *versus* subordinate juvenile Atlantic salmon in presence or absence of a competitive rainbow trout.

**Principal Findings:**

Adding the trout competitor resulted in dominant and subordinate salmon being more similar, both behaviourally and at the level of brain gene transcription patterns. Genes for which transcription levels differed between dominant and subordinate salmon in the absence of exotic trout were mainly over-expressed in dominant salmon and included genes implicated in protein turnover, neuronal structural change and oxygen transport.

**Conclusions/Significance:**

Our study provides one of the few examples demonstrating a close interplay between behavioural plasticity and gene transcription, therefore contributing to the understanding of the molecular mechanisms underlying these processes in an ecologically relevant context.

## Introduction

Biological invasion of species outside their native range is among the most important factors contributing to the ongoing biodiversity crisis [1]–[2]. Exotic species have strong ecological and evolutionary effects on invaded ecosystems [3]–[5]. Notably, behavioural changes in native populations facing invasion by exotic species have been reported in several taxa [6]. Rainbow trout (*Oncorhynchus mykiss*) are native to tributaries of the Pacific Ocean in Asia and North America, but have been introduced for food or sport in many locations throughout the world [7]–[8]. Both species display high microhabitat overlap in the wild, and it has recently been shown that competition imposed by the exotic rainbow trout strongly disrupted dominance hierarchies within groups of native Atlantic salmon (*Salmo salar*), as well as the phenotypic correlation between several behaviours [9]. Juvenile salmonids are territorial and form distinct social hierarchies both in the wild and when reared in captivity [9]–[10]. Identifying the genetic and molecular actors contributing to the plastic loss of dominance hierarchies previously reported in Atlantic salmon [9] is of fundamental interest in behavioural ecology [11]–[12], behavioural physiology and behavioural genetics [13].

Research in behavioural physiology and genetics has allowed the identification of several candidate genes and endogenous molecules modulating aggressive, territorial or dominance-related behaviours (reviewed in [14]). In salmonid fishes, the physiological causes and consequences of social status have been the subject of considerable research (reviewed in [15]). Modulation of brain monoaminergic activity (of neurons that secrete the monoamine neurotransmitters dopamine, norepinephrine and serotonin) by social interactions is generally seen as the basis for behavioural differences between fish of high and low social status [16]. Chronically high plasmatic levels of the corticosteroid hormone cortisol were repeatedly observed in socially defeated animals [10] and constitute evidence of chronic stress in subordinate fish [15]; this chronic stress could be related to many of the adverse physiological consequences of social subordination. Moreover, the so-called “challenge hypothesis” gives a central role to androgens (mainly testosterone and 11-ketosterone in fish) in the establishment of social hierarchy following contact among conspecifics [17]–[18], [11]. Neuropeptides of the vasotocin family (in fish: arginine vasotocin) and oxytocin-like peptides (in fish: isotocin) also have a role in behavioural plasticity, including plasticity of aggressive behaviour [19]. Other compounds associated with aggressive behaviour include nitric oxide [20], GABA [21], somatostatin [22], histamine, noradrenaline as well as several growth factors (neurotrophins), signalling proteins and metabolic enzymes (for a more exhaustive list, consult [14]).

Current knowledge may well represent only “the tip of the iceberg” of the complex architecture that controls aggressive behaviours [23]. Researchers recently used microarrays, which can track thousands of genes at once, to identify genes transcribed at different levels in the brains or whole bodies of animals from selected highly aggressive *versus* poorly aggressive strains or from dominant *versus* subordinate animals within strain (rainbow trout: [24], *Drosophila*: [23], [25], cichlids: [26]). Three of these studies identified several hundred differentially transcribed candidates from which genes previously identified as implicated in aggressive behaviour were conspicuously missing but in which genes implicated in functions such as energy metabolism, protein synthesis and even muscular contraction were over-represented [23]–[25]. In contrast, four candidates, including arginine vasotocin, were identified in the study of cichlid fishes [26].

The present study aimed at identifying genes regulating behaviours related to social dominance but also at understanding the association between gene expression and behavioural plasticity in the ecological context of species invasion. Hence, we compared, using a 16 006-gene salmonid microarray, the genome-wide gene transcription profiles of dominant *versus* subordinate juvenile Atlantic salmon in the presence or absence of a rainbow trout (exotic competitor) to test whether gene expression differences would reflect the plastic loss of dominance hierarchies in juvenile Atlantic salmon competing with rainbow trout. Particularly, we tested the hypotheses that (i) social hierarchies within pairs of Atlantic salmon changed in the presence of rainbow trout and (ii) changes in gene expression correspondingly occurred.

## Methods

### Behavioural experiment and analysis

#### Experimental design

We used young-of-the-year (YOY) Atlantic salmon and rainbow trout caught by electrofishing in the Malbaie River (Québec, Canada, 47°67′N; 70°16′W). In the sympatric section of the river, both species occupied similar macro-habitats and micro-habitat overlap increased as fish grew (see [9] for more details). Atlantic salmon were sampled in locations where rainbow trout are not present (i.e. above a human-controlled fish ladder) to avoid potential effects of previous encounters with rainbow trout. We selected juvenile salmon and trout of similar size to avoid confounding the effects of size and species (see [9]). In September 2005, Atlantic salmon and rainbow trout were transferred from Malbaie River to the laboratory. They were reared in separate holding tanks and fed *ad libitum* with commercial fish food pellets before experiments started.

**Figure 1:**
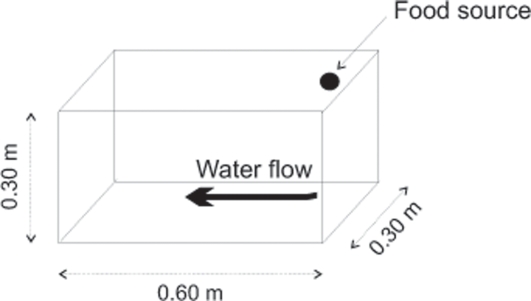
Experimental setting used to test the behaviour of Atlantic salmon in two competitive contexts. In total, twelve artificial channels were used simultaneously for the behavioral tests. The food was dispensed at a single fixed point and we recorded: (1) the position of each fish relative to the food source and (2) the time each fish spent being active.

Behavioural experiments were all performed simultaneously using 12 artificial channels made of transparent Plexiglas (Fig. 1). The channels and apparatus (i.e., water depth and velocity, water temperature, luminosity, etc.) are fully described in [9]. The only difference was the length of each channel (here, each was 0.60 m long, 0.30 m wide and 0.30 m deep, Fig. 1). Food rations (0.3 g artificial pellets) were manually dispensed each morning at a fixed food source, i.e. the upstream end of the channel (see Fig. 1). Twenty-four immature Atlantic salmon were visually selected from the holding tank to constitute twelve pairs of fish of similar size (mean fork length±SD: 66.20 mm±5.19 mm). No length differences were detected between treatments (mean fork length, ANOVA, F(1,22) = 0.07, p = 0.78). Each salmon was anaesthetized, measured and individually marked (Visible Implant Elastomer tags, Northwest Marine Technology, Shaw Island, Washington) before being released in the aquaria.

The behavioural experiment was performed in three steps. First, following introduction of the fish in the aquaria, the status of dominance (*i.e.*, subordinate or dominant) of individuals within each salmon pair was evaluated. Dominance was measured following the methodology described by Sloman et al. [27]. A mean behaviour score for position from the food source, food acquisition and social interaction was calculated for each fish during the first four days of the experiment (see [27] for more details). The fish with the highest score in a given pair was considered the dominant of that pair. At the end of these four days, the hierarchy was stable within each pair. Second, during the next three days we performed behavioural observations to characterise the behaviour of dominant and subordinate salmon in the absence of competing rainbow trout. Each channel was observed for 5 min each morning, directly after feeding the fish. We measured two behaviours: (1) the position of each fish relative to the food source and (2) the time each fish spent being active. A fish was considered as being active when it was out of a refuge, facing the current, and propped up on its pectoral fins. During these observations, aggressive acts were sparse and were not recorded. Third, after this seven-day period, one additional competitor was added to each of the aquarium. We added one rainbow trout in six channels (interspecific competition treatment) and one Atlantic salmon in the six other channels (intraspecific competition treatment). These supplementary fish were chosen haphazardly from the stock. This substitutive design allowed us to evaluate the effect of competition by the exotic species relative to an equivalent level of intraspecific competition and to maintain the same fish density in both treatments. The rainbow trout did not significantly differ in size from the Atlantic salmon we added (two-tails t-test, t = −0.63, p = 0.538). After a two day acclimation, we recorded the behaviour of members of each pair (previously identified as dominant and subordinate) in presence of a competing rainbow trout or of a third salmon (following the approach described above).

#### Statistical analysis

During the experiment, one subordinate fish died in the intraspecific competition treatment; the number of replicates was then five instead of six for this treatment. To evaluate whether the social hierarchies within pairs of Atlantic salmon changed after the addition of rainbow trout, we compared the behavioural repertory of dominant and subordinate Atlantic salmon before (second step of the experiment) and after the addition of a competitor (rainbow trout or Atlantic salmon, third step of the experiment) in the aquaria. Instead of analysing each behavioural variable independently, we used a multivariate analysis of variance with repeated measures (MANOVAR, [28]–[29]) to test for behavioural changes between the second and the third step of the experiment. The dependent variables were the position of each fish relative to the food source (log transformed) and the time each fish spent being active (arcsine transformed). We used the “dominance rank” (dominant or subordinate) and the “competitive treatment” (interspecific or intraspecific competition) as independent variables. The “period of observation” (before or after the addition of a competitor) was the within-subject factor (*i.e.*, the repeated measure). All possible interaction terms were considered.

### Transcriptomic experiment

#### RNA extraction, labelling and cDNA hybridisation

Following the behavioural experiment, all fish were anaesthetised and whole brains were taken from both salmon of each initial pair. Brains were immediately frozen in liquid nitrogen, and later homogenised individually in TRIZOLReagent (Invitrogen, San Diego) using a Diax 100 homogeniser (Heidolph instruments). Total RNA was extracted as previously described [30]–[31]. For each sample, 5 µg total RNA was retro-transcribed and labelled using Genisphere Array 350 3DNA array detection kits and the Superscript II retro-transcriptase (Invitrogen, San Diego) according to the manufacturer's instructions. Transcription profiles of six dominant and six subordinate salmon were contrasted on six microarrays. Three of the salmon pairs considered in the microarray experiment had faced competition by an exotic rainbow trout in the last part of the behavioural experiment while a third salmon had been added in the aquaria of the other three pairs (see above). The cDNA microarrays used here were obtained through the consortium Genomic Research on All Salmon Project (cGRASP, available from Ben F. Koop, bkoopuvic.ca), and contain 16,006 salmonid cDNA clones, the great majority of which (99.8%) are not replicated. However, a same gene can be represented on the chip by several different cDNA clones [32].

#### Signal detection, data preparation and statistical analysis

Signal detection and data preparation was done as previously reported [30]. Spots with mean intensities for both the dominant or subordinate categories smaller than the mean intensity of control empty spots plus twice its standard deviation or with a coefficient of variation above one for either the dominant or subordinate categories were removed from the analysis, leaving 5142 and 5124 cDNA clones to be analysed for the interspecific and intraspecific competition experiments, respectively. Gene transcription data from the interspecific and intraspecific competition experiments were analyzed in two separate ANOVA using the MAANOVA R package [33]–[34] The ANOVA model included in each case the “array” term as a random term and the “social rank” (dominant or subordinate) and “dye” terms as fixed terms. A permutation-based F-test (Fs, with 1000 permutations) was then performed and restricted maximum likelihood was used to solve the mixed model equations. Specifically, R/MAANOVA recreates a null distribution of the data by randomly permuting the columns in the datasets in order to calculate the permutation-based p-value. Q-values were calculated from the permutation based p-values using the Q-value R package [35]. The Q-value of a test measures the proportion of false positives incurred (false discovery rate or FDR) when that particular test is called significant. Hierarchical clustering analysis between genes and between treatments was run using the GeneSight 3.5 software (BioDiscovery).

## Results

### Behavioural experiment

**Figure 2:**
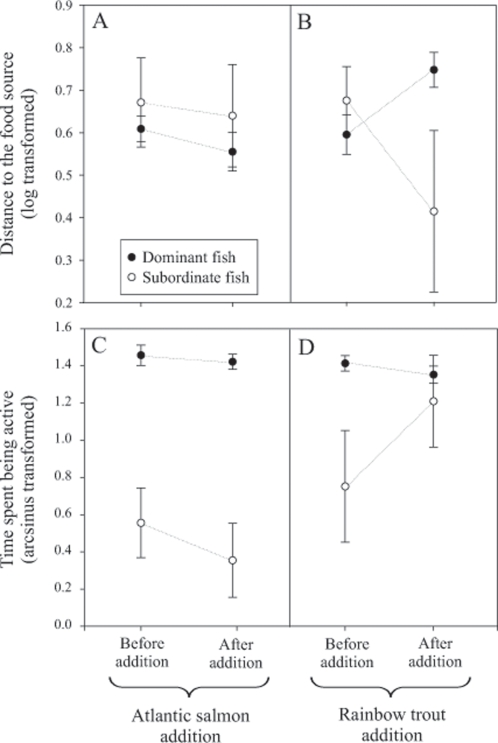
Behavioural characteristics of juvenile Atlantic salmon in two competitive contexts. The behavioural characteristics of dominant (black dots) *vs.* subordinates (white dots) Atlantic salmon (*Salmo salar*) before and after the addition of either intraspecific (A and C), or interspecific (the exotic rainbow trout; B and D) competitors. The distance each fish was from the food source (upper panel), and the time each fish spent being active (lower panel) were used to characterize the behaviour of dominant and subordinate salmon.

As previously reported, rainbow trout strongly disrupt the social hierarchy between subordinate and dominant juvenile Atlantic salmon (Table 1, Figures 2A–2D) [9]. Indeed, in the absence of rainbow trout, dominant and subordinate salmon significantly differed in the behaviours they displayed, with dominant fish being closer to the feeding source (Figures 2A–2B) and also more active (Figures 2C–2D). After rainbow trout were added into the system, subordinate and dominant fish tended to be behaviourally more similar one to each other, particularly in time spent being active (Figure 2D). As for the distance to the feeding source, the situation was almost reverse since subordinate salmon tended to be closer to the feeding source (Figure 2B). However, it is worth noting the huge variation observed for subordinate salmon, which may suggest a stronger interaction between individuals. When an Atlantic salmon was added into the aquaria instead of a rainbow trout, neither the dominant nor the subordinate fish were affected by this additional competitor (Figures 2A, 2C), thus supporting the idea that the effect of rainbow trout on the behaviour of Atlantic salmon was highly species-specific (see also [9]). Indeed, dominant fish remained closer to the feeding source than subordinate fish (Figure 2A) and were still more active (Figure 2C).

**Table 1:** Results of a MANOVAR used to evaluate whether behavioural changes of dominant and subordinate Atlantic salmon occurred when rainbow trout or Atlantic salmon are added as competitors.

	Wilks'λ	F-value	d.f.	P-value
Independent variables				
Dominance rank	0.463	9.905	2,17	0.001
Competitive Treatment	0.888	1.065	2,17	0.366
Period of observation	0.892	1.044	2,17	0.373
Dominance*Treatment	0.709	3.488	2,17	0.053
Dominance*Treatment	0.607	5.485	2,17	0.014
Treatment*Period	0.581	6.154	2,17	0.009
Dominance*Treatment*Period	0.391	13.191	2,17	<0.0001

### Transcriptomic experiment

**Figure 3:**
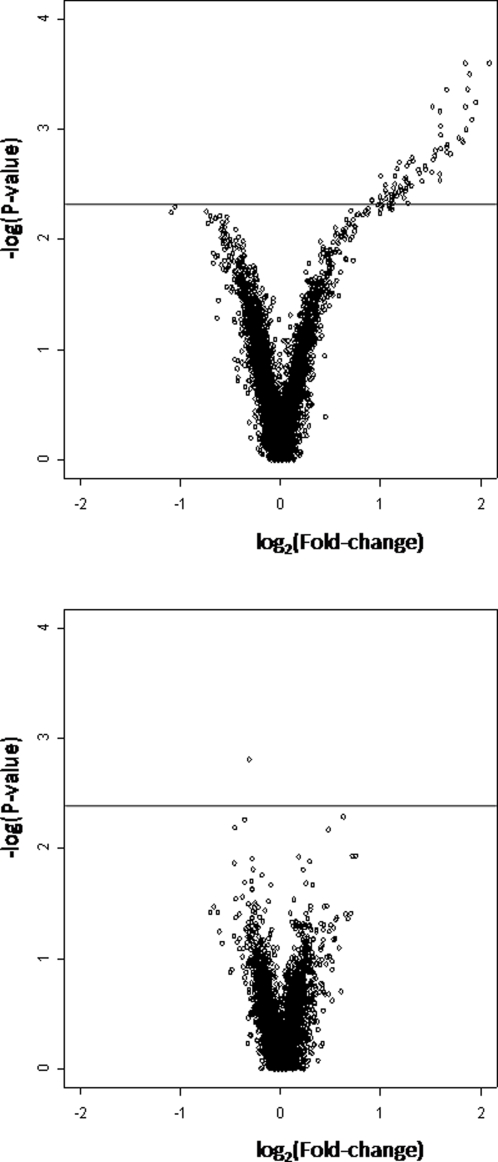
Genome-wide gene transcription profiles in brains of juvenile Atlantic salmon in two competitive contexts. In an ANOVA comparing (A) dominant and subordinate salmon in the absence of rainbow trout and (B) dominant and subordinate salmon in presence of a rainbow trout, these volcano plots present the significance (-log(P-value), Y-axis) of the observed difference in transcription for each of the 5142 detected gene against the magnitude of this difference (log_2_(average fold change), X-axis). Positive log_2_(average fold change) values represent genes over-transcribed in the brain of dominant juvenile salmon while negative log_2_(average fold change) values represent gene under-transcribed in dominant versus subordinate salmon.

Figure 3 shows that, for any given significance threshold, substantially more genes are differentially transcribed between dominant and subordinate Atlantic salmon in the absence (Figure 3A) than in the presence of competitive rainbow trout (Figure 3B). This result is not associated with increased experimental error in the interspecific competition experiment, since both experiments were carried out at the same time, by the same person using the same material. Moreover, the average coefficient of variation (CV) of the normalized hybridization signals for all genes was smaller for the interspecific than for the intraspecific competition situation (0.235 and 0.240, respectively).

Only one gene showed significant transcription level differences between dominant and subordinate salmon that had been exposed to a rainbow trout at the P<0.005 significance threshold, which is less than expected by chance alone. This gene was hence considered as a false positive and therefore not interpreted any further. By contrast, the comparison of dominant and subordinate fish in a purely intraspecific competition context revealed more significant differences than expected by chance (73 significant cDNA clones, while 25 are expected by chance alone at the P<0.005 significance threshold). This absolute value must however be interpreted cautiously since estimation of the expected number of false positives from the total number of spots analyzed might be misleading as a same gene can be represented by several spots (same or different ESTs) and the expression of many genes is expected to be correlated, which cannot be accounted for in the analysis. It is also noteworthy that all of the differentially expressed transcripts at P<0.005 appeared over- rather than under-transcribed in dominant individuals which corroborates the results of previous transcriptomic experiments which have documented gene transcription differences implicated in aggressive behaviour [23], [25].

Table 2 presents the 27 different gene products corresponding to the 73 cDNA clones which showed significant transcription level differences in the brains of dominant and subordinate juvenile salmon (P<0.005) in the intraspecific experiment. Given the small sample size and the inter-individual variability of the detected signal, these candidates still have non-negligible chances of being false positives (q-values between 0.159 and 0.196). Six candidates were marked as “unknown”, since the corresponding cDNA clone sequence did not generate any BLAST hits with e-values <1×10^−15^ and an informative name during the array annotation process. The functions of the remaining candidates are discussed in the following section.

**Table 2:** Gene products corresponding to the 73 cDNA clones which showed significant transcription level differences in the brain of dominant and subordinate juvenile salmon (P<0.005) in the absence of rainbow trout.

Gene product or cDNA clone	P-value	Q-value	Fold change	cDNA clone
**Protein degradation**				
Ubiquitin-conjugating enzyme E2G 2	2.5×10^−04^	0.159	4.0	1
Ubiquitin carboxyl-terminal hydrolase isozyme L1	3.4×10^−03^	0.195	2.6	1
Proteasome subunit alpha type 1	4.7×10^−03^	0.196	2.2	1
**Oxygen transport**				
Hemoglobin alpha	2.5×10^−04^	0.159	4.4	22
Hemoglobin beta	4.4×10^−04^	0.174	4.1	18
Hemoglobin epsilon	6.3×10^−04^	0.181	3.7	6
**Immunity-related**				
Peptidyl-prolyl cis-trans isomerase B	8.2×10^−04^	0.193	4.0	2
MHC class I antigen pseudogene and proteosome subunit LMP7/PSMB8	3.2×10^−03^	0.195	2.9	1
**Apoptosis-related**				
Caspase 8	1.0×10^−03^	0.193	3.8	1
TGFB-inducible early growth response protein 2	2.3×10^−03^	0.194	3.2	1
**Signal transduction**				
Tumor protein D53	1.4×10^−03^	0.193	3.5	1
Guanine nucleotide-binding protein	3.0×10^−03^	0.195	2.7	1
**Transcription/protein synthesis**				
DNA-directed RNA polymerase	2.3×10^−03^	0.194	2.8	2
60S ribosomal protein L28	3.8×10^−03^	0.196	2.4	2
**Actin cytoskeleton organisation**				
Actin-related protein 1 homolog B	2.9×10^−03^	0.195	2.9	1
Kelch-like protein 1	4.5×10^−03^	0.196	2.1	1
**Miscellanous**				
Midasin	1.9×10^−03^	0.193	3.4	1
Myosin regulatory light chain 2	4.2×10^−03^	0.196	2.4	1
Brain lipid-binding protein	4.6×10^−03^	0.196	2.1	1
Collagen alpha 2(I)	4.8×10^−03^	0.196	2.5	1
Biotinidase	4.9×10^−03^	0.196	2.2	1
**Unknown function**				
CA053773 UNKNOWN	1.3×10^−03^	0.193	3.6	1
CA060279 UNKNOWN	1.3×10^−03^	0.193	3.6	1
CA037818 UNKNOWN	1.6×10^−03^	0.193	3.6	1
CA061786 UNKNOWN	2.0×10^−03^	0.193	3.5	1
CB501353 UNKNOWN	4.3×10^−03^	0.196	2.3	1
CK991021 UNKNOWN	4.9×10^−03^	0.196	2.1	1

Permutation-based P-values from the ANOVA are presented, as well as the corresponding Q-values, the average fold change in gene transcription level and the number of distinct significant cDNA clones corresponding to each gene product. In cases where a gene was represented by more than one significant cDNA clone, only data from the most significant cDNA clone is presented.

**Figure 4:**
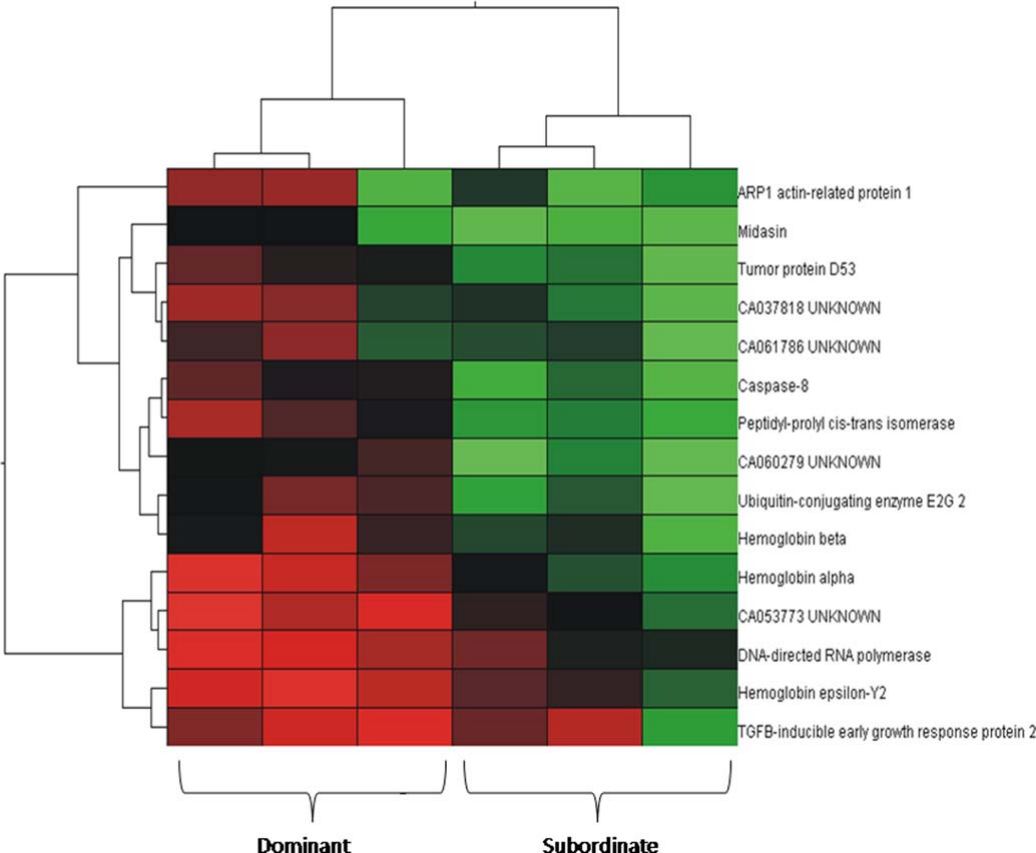
Differences in transcript abundance in the brains of subordinate and dominant juvenile Atlantic salmon. Graphical representation of the differences in transcript abundance in the brains of three subordinate (right) and three dominant (left) juvenile salmon in the absence of rainbow trout. For each individual, the normalized transcription level is represented for the 15 most significantly differentially expressed genes by a coloured box (red: high expression, green: low expression). Only the data from the most significant cDNA clone were considered in the case of genes represented by several significant cDNA clones. Hierarchical clustering of gene expression data by gene and by experiment is shown as a horizontal and a vertical tree, respectively. The trees represent relationships between expression patterns, with branch length indicative of the magnitude of the differences between these patterns across genes or samples.

Figure 4 shows that the normalized hybridization signals for the 15 most significantly differentially expressed non-redundant genes can be used to accurately separate dominant and subordinate individuals in the intraspecific experiment. Such sorting was not possible for pairs exposed to the trout competitor (not shown). We also performed a hierarchical clustering on the data of the 5155 significantly expressed cDNA in the intraspecific competition context (not shown). As we expected, the gene transcription data did not group by control and treatment samples, but rather by microarray. Hence, the minority (73 cDNA clones) of genes of which the transcription level seem to differ between dominant and subordinate salmon had a neglectable weight when clustering considering all (5155 cDNA clones) expressed sequences, given the experimental variance associated with the microarrays themselves. High experimental variance associated with the microarrays and the dyes, notably, is not specific to this work but is a general property of microarrays experiments (see, for instance, [36]), making it important to consider these sources of variance in the ANOVA model.

## Discussion

This study identified genes implicated in behavioural differences related to social dominance, which contributes to the understanding of the relationship between gene expression and behavioural plasticity in the context of competitive interactions between native and invasive species. Namely, our results provide evidence for the differential transcription of 27 different genes between dominant and subordinate salmon. Additionally, the greater degree of similarity in the behaviour of subordinant and dominant salmon in presence of the exotic competitor (see also [9]) reflected the paucity of transcriptional differences observed between subordinate and dominant salmon after the introduction of an interspecific competitor in the gene transcription experiment. Thus, the presence of the exotic competitor (rainbow trout) apparently suppressed most of the transcriptional differences between dominant and subordinate salmon. Some of the suppressed differences might represent changes causing the loss of dominance hierarchy, whereas others might be a consequence of it; this study cannot disentangle such causal links. Yet, the identification of genes differentially regulated between dominant and subordinate salmon in absence but not in presence of trout is a first step towards clarifying the molecular mechanisms associated with the plastic breakdown of social hierarchies. In particular, co-regulated and functionally related candidate genes could help identifying molecular actors implicated in the differences at the behavioural and transcriptional levels. Yet hierarchical clustering by differentially expressed genes (Figure 4) and scanning of the literature for their potential regulatory relations did not reveal any conspicuous pattern that would point to one or a few key regulator genes in the present study.

Admittedly, the use of a microarray not specific for brain cDNA to analyse gene expression in the brain is not without limitations [23], [25], [37]. For instance, the most obvious candidate genes for aggressive behaviour (see Introduction) were not represented on the microarray we used, which was not specific for brain tissue (4 of the 33 salmonid cDNA libraries used for constructing the microarray were from brain tissue), and were therefore not among the candidates identified here. This can also generate results which seem puzzling at first glance. For example, we observed differential expression of several cDNA clones representing three different globin genes (Table 2). Differential haemoglobin chain expression in brain tissue between distinct phenotypes or populations, including salmonids, has been reported previously [25], [37]–[38]. Hence, higher brain expression of both alpha- and beta-globin mRNA was observed in Atlantic salmon reared in laboratory conditions compared to fish reared in natural streams [37]. Unlike their mammalian counterparts, mature fish erythrocytes are nucleated and can synthesise haemoglobin while circulating in the blood [39]–[40]. This raises the hypothesis that, in fish, increased transcription of haemoglobin genes could occur within the fish nucleated red blood cells and contribute to or be a consequence of a dominant social status.

Three genes implicated in protein degradation were over-transcribed in dominant *versus* subordinate salmon (Table 2). The expression of one of these, *ubiquitin carboxyl-terminal esterase L1*, is highly specific to neurons (and reproductive organs) in mouse and may insure ubiquitin stability within neurons [41]. This and the over-transcription of genes implicated in transcription and translation (Table 2) might suggest increased overall protein turnover in the brains of dominant fish. Differential transcription of several genes implicated in protein degradation as well as of ribosomal proteins as also been observed in *Drosophila* strains selected for aggressive behaviour (see [25], but they did not specify in which strain individual genes were over-transcribed). Protein degradation and synthesis were also mentioned among the main functional categories of genes showing contrasting transcription levels between dominant and subordinate rainbow trout [24]. However, the authors did not specify the identity of the genes and direction of the over-transcription [24]. A fourth gene over-transcribed in dominant fish and implicated in protein degradation (Table 2) encodes a proteasome subunit critical for class I antigen presentation in mouse (proteasome subunit LMP7, [42]). This gene was therefore classified in the “immunity-related” category rather than in “protein degradation”. Interestingly, the gene encoding kelch-like 1, a protein primarily expressed in brain where it is hypothesised to have a role in the organisation of the actin cytoskeleton [43], was also over-expressed in dominant salmon (Table 2). While behavioural plasticity is expected to be initially based on changes in neuronal activity and excitability as well as endocrine responses, subsequent changes in brain and behaviour (e.g. memory formation) are expected to result from structural and physiological changes in neurons [13]. Also, it has recently been found that neuron proliferation was reduced in subordinate *versus* dominant rainbow trout [44]. In this context, over-expression of *kelch-like 1* in dominant salmon could be implicated in increased structural changes in neurons or in organizing newly formed neurons. In the same way, brain lipid-binding protein, also over-expressed in dominant *versus* subordinate salmon (Table 2), is a fatty acid-binding protein that was suggested to play a role in neuronal and glial cell differentiation [45]. Admitedly, however, results for individual genes of interest would have to be confirmed in future studies, given the relatively small sample sizes available for these experiments.

To conclude, our study provides one of the few examples demonstrating a close interplay between behavioural plasticity and changes in gene expression in an ecologically relevant context. Behavioural plasticity is a key mechanism for animals facing rapid ecological changes such as species invasion [46], and molecular mechanisms underpinning this plasticity are actually not completely understood [11]. Our study therefore contributes substantially to this common effort of clarifying the molecular mechanisms of behavioural plasticity. Moreover, our results (see also [9]) provide evidence for the influence of an introduced competitor on salmon intra-specific competitive interactions. Since such intra-specific interactions are known to play a role in the evolution of salmon reproductive strategies, this raises the hypothesis that the introduction of rainbow trout could impact on the evolution of salmon populations for such traits. Indeed, two major male reproductive strategies co-exist in Atlantic salmon (anadromous dominant males and sexually precocious sneakers) which appear to be partly heritable [47] and are linked to the dominance status of individuals at the juvenile stage [48]–[49]. In the context of game theory [50], the virtual suppression of dominance hierarchies in salmon by exotic rainbow trout may then disrupt the evolutionarily stable strategy (ESS) of the two male reproductive strategies in salmon. Moreover, the identification of genes for which the transcription level is altered by intra- and inter-specific interactions provides candidates towards a better understanding of the molecular mechanisms that could be involved in the evolution of salmon reproductive strategies.
